# Targeting MAPK Signaling: Loureirins A and B from *Dracaena Loureiri* Inhibit Epithelial–Mesenchymal Transition and Invasion in Non-Small Cell Lung Cancer Cell Lines

**DOI:** 10.3390/life15030396

**Published:** 2025-03-03

**Authors:** Xiaomin Huang, Punnida Arjsri, Kamonwan Srisawad, Sonthaya Umsumarng, Supachai Yodkeeree, Pornngarm Dejkriengkraikul

**Affiliations:** 1 Department of Biochemistry, Faculty of Medicine, Chiang Mai University, Chiang Mai 50200, Thailand; xiaomin_huang@cmu.ac.th (X.H.); punnida.dream@gmail.com (P.A.); kamonwan.sri@cmu.ac.th (K.S.); supachai.y@cmu.ac.th (S.Y.); 2 Anticarcinogenesis and Apoptosis Research Cluster, Faculty of Medicine, Chiang Mai University, Chiang Mai 50200, Thailand; 3 Faculty of Veterinary Medicine, Chiang Mai University, Chiang Mai 50200, Thailand; sonthaya.u@cmu.ac.th; 4 Center for Research and Development of Natural Products for Health, Chiang Mai University, Chiang Mai 50200, Thailand

**Keywords:** loureirin A, loureirin B, anti-invasion, non-small cell lung cancer, MAPK pathway

## Abstract

Metastasis remains the leading cause of death among patients with non-small cell lung cancer (NSCLC), emphasizing the urgent need for safer and more effective therapeutic options. Mitogen-activated protein kinase (MAPK) pathways play a crucial role in regulating EMT, migration, and invasion in NSCLC. Targeting these molecular mechanisms has become a key strategy in inhibiting NSCLC metastasis. Loureirin A and Loureirin B, flavonoids derived from the Thai traditional herb *Dracaena loureiri*, have shown potential pharmacological effects; however, their roles in NSCLC metastasis remain unexplored. This study aimed to elucidate the mechanisms by which Loureirin A and Loureirin B suppress EMT, migration, and invasion in NSCLC cells via the MAPK signaling pathway. The sulforhodamine B (SRB) assay showed that Loureirin A and Loureirin B, at concentrations ranging from 0 to 140 μM, were non-toxic to both A549 and H1299 cells. Additionally, Loureirins A and B exhibited no cytotoxic effects on primary human dermal fibroblast cells and did not induce hemolysis in red blood cells (RBCs). The wound-healing and trans-well assays were used to evaluate the anti-migratory and anti-invasion properties of Loureirin A and Loureirin B in NSCLC cell lines. Gelatin zymography was employed to investigate the activity of MMP-2 (gelatinase A) and MMP-9 (gelatinase B), while Western blot analysis was used to examine the expression of EMT markers and invasive proteins, and the phosphorylation of MAPK signaling molecules. Our results demonstrate that both Loureirin A and Loureirin B significantly suppressed the migration and invasion of A549 and H1299 cells. These compounds suppressed the activity of matrix metalloproteinases MMP-2 and MMP-9 and downregulated the expression of key invasive proteins including uPA, uPAR, and MT1-MMP. Additionally, they effectively suppressed the expression of EMT markers such as N-cadherin, Vimentin, and Fibronectin. Mechanistically, Loureirin A and Loureirin B inhibited the MAPK signaling pathway by downregulating the phosphorylation of ERK, JNK, and p38 proteins. In conclusion, these findings demonstrate that Loureirin A and Loureirin B exhibit potent anti-invasive properties and no cytotoxic effect on NSCLC cell lines, suggesting their potential as promising candidates for anti-cancer drug development. Furthermore, they may pave the way for the exploration of combination therapies with other anti-cancer drugs for clinical translation.

## 1. Introduction

Non-small cell lung cancer (NSCLC) represents a major global health challenge, accounting for the highest number of cancer-related deaths worldwide [1,2,3]. As the most prevalent subtype of lung cancer, comprising approximately 85% of all cases, NSCLC is often diagnosed at advanced stages, when the disease has already progressed significantly [3,4]. A key barrier to its effective treatment is metastasis, the dissemination of cancer cells from the primary tumor to distant organs. Despite advancements in molecular-targeted therapies and immunotherapies, the high migratory and invasive potential of NSCLC cells continues to hinder therapeutic success, contributing to poor prognoses [5,6]. Metastasis frequently results in local recurrence or the formation of secondary tumors, further limiting improvements in the 5-year survival rates of NSCLC patients [7,8,9]. The treatment used for NSCLC depends on the cancer stage and its molecular characteristics, with the options including surgery, chemotherapy, targeted therapy, immunotherapy, and radiation [10]. Most commonly used anti-cancer drugs for NSCLC target growth-factor receptors (e.g., gefitinib, erlotinib) or involve immunotherapy (e.g., pembrolizumab, nivolumab) [11]. However, challenges such as effectively targeting cell signaling pathways and the metastatic process drive ongoing research into combination therapies and novel strategies to enhance the efficacy and minimize the side effects of NSCLC.

Epithelial–mesenchymal transition (EMT) is a crucial biological process that is implicated in cancer metastasis [12]. During EMT, epithelial cancer cells lose cell–cell adhesion and acquire mesenchymal-like properties, enhancing their motility and invasiveness [13]. This process is characterized by the decreased expression of epithelial markers, such as E-cadherin, and the increased expression of mesenchymal markers, including N-cadherin, vimentin, and fibronectin [14]. Furthermore, proteins like matrix metalloproteinases (MMPs), urokinase-type plasminogen activator (uPA), urokinase-type plasminogen activator receptor (uPAR), and membrane type 1 matrix metalloproteinase (MT1-MMP) facilitate extracellular matrix degradation, a critical step in cancer cell invasion and metastasis [15,16]. Intracellular signaling pathways, particularly the mitogen-activated protein kinase (MAPK) pathway, play a pivotal role in regulating EMT, migration, and invasion [17,18,19]. Consequently, targeting these molecular mechanisms has become a focal point in NSCLC metastasis research.

Recent studies have highlighted the potential of phytochemicals from medicinal herbs to suppress tumor progression [20,21]. Both in vitro and in vivo research has demonstrated that these compounds can modulate signaling pathways to inhibit the growth and spread of NSCLC cells [22,23,24]. Among such phytochemicals are Loureirin A and Loureirin B, flavonoids derived from *Dracaena loureiri*, a traditional Thai medicinal plant renowned for its anti-inflammatory and therapeutic properties [25,26]. Loureirin A has been shown to inhibit platelet aggregation [27], reduce oxidative stress, and suppress inflammatory responses by downregulating pro-inflammatory mediators, including interleukins and tumor necrosis factor-alpha (TNF-α) [28]. Similarly, Loureirin B has exhibited anti-inflammatory activity and promoted wound-healing by modulating fibroblast proliferation and extracellular matrix remodeling [29,30]. Both compounds also possess anticoagulant and antifibrotic properties, suggesting their potential in managing various pathological conditions [27,31,32].

Despite their well-documented anti-inflammatory and tissue repair effects, the roles of Loureirin A and Loureirin B in cancer progression remain largely unexplored, particularly their anti-migration and anti-invasion potential in NSCLC. Addressing this knowledge gap is critical to understanding whether these compounds can effectively inhibit EMT, migration, and invasion, which are the key processes driving cancer metastasis. In this study, we investigated the anti-migration and anti-invasion effects of Loureirin A and Loureirin B in NSCLC cell lines (A549 and H1299 cells). Specifically, we aimed to elucidate the mechanisms by which these compounds suppress EMT, migration, and invasion, focusing on their modulation of the MAPK signaling pathway. Our findings demonstrate that Loureirin A and Loureirin B inhibit EMT-associated markers, suppress the activity of MMP-2 and MMP-9, and reduce the expression of invasive proteins. Due to modulating the MAPK pathway, these compounds represent potential therapeutic agents that can inhibit NSCLC migration and invasion.

## 2. Materials and Methods

### 2.1. Chemicals and Reagents

Loureirin A (Cat. No. CFN92766) and Loureirin B (Cat. No. CFN98173) were obtained from MedChemExpress company (Monmouth Junction, NJ, USA). Dulbecco’s Modified Eagle Medium (DMEM) (Cat. No. 12800-017), 10× trypsin enzyme (Cat. No. 15090-046), 100× penicillin-streptomycin antibiotics (Cat. No. 15140-122), and fetal bovine serum (FBS) (Cat. No. A5256701) were purchased from Gibco BRL (Grand Island, NY, USA). The sulforhodamine B (SRB) (Cat. No. S1402) reagent and primary antibody for anti-β-actin (Cat. No. A5316) were obtained from Sigma-Aldrich (St. Louis, MO, USA). Corning^®^ Matrigel^®^ Basement Membrane Matrix (Cat. No.356234) was obtained from Corning Life Science (Corning, NY, USA). For Western blot analysis, the RIPA lysis buffer, the protease inhibitor cocktail (Cat. No. 89901), the reagent of Coomassie PlusTM Protein Assay (Cat. No. 1856210), the enhanced chemiluminescence (ECL) reagent (Cat. No. RPN2236), and the Restore™ PLUS Western Blot Stripping Buffer (Cat. No. 46430) were obtained from Thermo Fisher Scientific (Rockford, IL, USA). The primary antibodies for Western blot analysis, used to detect invasive proteins (u-PA, u-PAR, and MT1-MMP) and EMT markers (fibronectin, N-cadherin, and vimentin) and MAPK signaling pathway (p-ERK1/2 (cat. no. 4377S), ERK1/2 (cat. no. 4696S), p-JNK (cat. no. 9255S), JNK (cat. no. 9252S), p-p38 (cat. no. 4631S), and p38 (cat. no. 9212S)) protein expression, and the secondary antibodies, namely goat anti-mouse (cat. no. 7076S) or rabbit (cat. no. 7074S) IgG conjugated with horseradish peroxidase, were obtained from Cell Signaling Technology (Beverly, MA, USA).

### 2.2. Cell Lines and Cell Culture

The cell line models used in this study were A549 (CCL-185™) and H1299 (CRL-5803™), both of which are human NSCLC cell lines. Both cell lines were purchased from the American Type Culture Collection (ATCC) (Manassas, VA, USA). A549 and H1299 cells were maintained in Dulbecco’s Modified Eagle Medium (DMEM). For the supplements, the cells were cultured with 10% FBS, penicillin (50 IU/mL), and streptomycin (50 μg/mL). They were cultured in an incubator at 37 °C with 5% CO_2_. The doubling times of A549 and H1299 cells were determined to be approximately 22 h and 18 h, respectively, which is consistent with a previous study [33].

Primary human dermal fibroblasts were aseptically isolated from an abdominal scar following a cesarean delivery performed at the surgical operation room of Chiang Mai Maharaj Hospital, Chiang Mai University, Chiang Mai, Thailand. The study was conducted under the ethical approval of the Medical Research Ethics Committee, Chiang Mai University (Study code: BIO-2567-0035). Fibroblast cells were isolated using a previously described protocol [34]. The cells were cultured in DMEM supplemented with 10% FBS, 2 mM L-glutamine, 50 U/mL penicillin, and 50 g/mL streptomycin and maintained in a humidified incubator at 37 °C with 5% CO_2_. The doubling time of human dermal fibroblast cells was approximately 60 h, consistent with a previous study [35].

### 2.3. Red Blood Cell (RBC) Hemolysis Assay

The effects of Loureirin A and Loureirin B on human red blood cells (RBCs) were assessed using a hemolysis induction assay following a previously described protocol [36,37]. Human blood samples were obtained from the Blood Bank Laboratory at Maharaj Hospital, Chiang Mai, Thailand. The samples were anonymized and could not be traced back to individual donors (study code: BIO-2567-0035, approved by the Medical Research Ethics Committee, Chiang Mai University).

Briefly, packed RBCs were diluted in 0.86% normal saline solution (NSS) to prepare a 5% RBC suspension. A total of 300 μL of the 5% RBC suspension was incubated with different concentrations (0–280 μM) of Loureirin A and Loureirin B at 37 °C for 4 h. NSS was used as the negative control, while 0.5% Triton X-100 served as the positive control. After incubation, the supernatant was collected by centrifugation at 5000 rpm for 5 min at room temperature, and hemoglobin concentrations were measured spectrophotometrically at 540 nm. The concentration of cell-free hemoglobin in each sample was calculated using a hemoglobin standard curve.

### 2.4. Cell Viability Assay

We performed a sulforhodamine B (SRB) assay to evaluate the effects of Loureirin A and Loureirin B on the viability of NSCLC cell lines, specifically A549, H1299, and primary human dermal fibroblasts cells [38]. The cell lines were plated at a number of 3 × 10^3^ cells/well into 96-well plates and incubated overnight. Loureirin A and Loureirin B were used to treat the cells at concentrations ranging from 0 to 350 μM, followed by incubation for 24 and 48 h. After incubation, we added 10% (*w*/*v*) trichloroacetic acid (100 μL) to each well, and the cells were incubated for 1 h at 4 °C. The wells were then washed thoroughly using gently flowing tap water. Once the wells were dried, an SRB solution (0.054% *w*/*v*) of 100 μL was added to each well, and the cells were left to incubate for 30 min at 25 °C. Subsequently, we removed the SRB dye and washed the cells three times with 1% (*v*/*v*) acetic acid in aqueous solution. Then, Tris-base solution, 10 mM, pH 10.5 was added to dissolve the SRB dye. Absorbance was assessed at 510 nm by a microplate reader, and cell viability was determined as a percentage relative to the control.

### 2.5. Wound-Healing Migration Assay

We performed a wound-scratch assay following an established protocol to assess the migratory ability of NSCLC cell lines [39]. A549 and H1299 cells were plated at a number of 2.5 × 10^5^ cells/well in 6-well plates and incubated overnight. After the cells reached 90–100% confluence, they were starved in 0.5% FBS DMEM for 18 h. A yellow pipette tip was used to create a wound on the cell monolayer in each well plate. Subsequently, Loureirin A and Loureirin B, at concentrations ranging from 0 to 140 μM, were added to the wells containing wounds which were then incubated for 24 h. Images were captured at the same fields at specified time intervals (0 and 24 h) using a phase-contrast microscope (Nikon Eclipse TS100, Nikon Instruments Inc., Tokyo, Japan) at 100× magnification. The ‘Wound Healing Size’ plugin in ImageJ 1.41 software was utilized to analyze the wound area and precisely outline the wound region for each experimental condition [40].

### 2.6. Trans-Well Invasion Assay

To assess the effects of the Loureirin A and Loureirin B compounds on the invasive abilities of A549 and H1299 cells, trans-well invasion assays were conducted following a previously established protocol [41]. Polycarbonate filters without polyvinylpyrrolidone and with 8 μm pore size (BD Biosciences, Franklin Lakes, NJ, USA) were utilized, and each filter was coated with 15 μg of Matrigel, which served as a basement membrane rich in extracellular matrix proteins. The upper sides of the trans-well inserts contained A549 and H1299 cells (5 × 10^4^ cells/well) and were cultured in DMEM supplemented with 0.1% FBS and varying concentrations of Loureirin A and Loureirin B compounds (0–140 μM). The lower chamber was filled with DMEM supplemented with 10% FBS, which acted as a chemoattractant. Invaded cells on the lower side of the trans-well filter were fixed with 95% ethanol (5 min) and then stained with 0.5% crystal violet dissolved in 20% methanol (30 min). The stained cells were observed under a phase-contrast microscope (Nikon Eclipse TS100, NIKON INSTRUMENT INC., Melville, NY, USA), and images were captured. To evaluate invasion, the cell-covered areas were analyzed using the ’Threshold’ tool in ImageJ software version 1.410 (https://imagej.nih.gov/ij/, accessed on 25 January 2025). The threshold settings were meticulously fine-tuned to differentiate cells from the background, ensuring precise and dependable measurements.

### 2.7. Gelatin Zymography Assay

A gelatin zymography assay was performed to evaluate the effects of Loureirin A and Loureirin B on MMP-2 and MMP-9 activity in A549 and H1299 cells. Gelatin, a denatured form of collagen and a natural extracellular matrix protein, was used as the substrate to assess the enzymatic activity of MMP-2 (gelatinase A) and MMP-9 (gelatinase B). Initially, A549 and H1299 cells were plated into six-well plates at a density of 2 × 10^5^ cells/well and left to incubate overnight. Subsequently, the cells were exposed to different concentrations of Loureirin A and Loureirin B (0–140 μM) and incubated for 24 h. Following incubation, the culture supernatant was collected for analysis. The gelatinolytic activity of secreted MMP-2 and MMP-9 in the conditioned medium was assessed using gelatin zymography. The MMP-9 (92 kDa) and MMP-2 (72 kDa) were separated using SDS-PAGE on a 10% polyacrylamide gel containing 0.1 mg/mL gelatin under non-reducing conditions. After that, the gel was rinsed twice with 2.5% Triton X-100 (30 min) and then incubated in an activation buffer (50 mM Tris-HCl, 200 mM NaCl, and 10 mM CaCl_2_, pH 7.4) at 37 °C for 24 h. The gel was then stained with 0.1% Coomassie Brilliant Blue R-250 and decolorized using a solution of 10% acetic acid and 50% methanol. Gelatinolytic activity was quantified by analyzing band intensity with IMAGE J software version 1.410.

### 2.8. Western Blot Analysis

For Western blot analysis, Loureirin A and Loureirin B were used to treat the A549 and H1299 cells at concentrations ranging from 0 to 140 μM for 24 h. We used RIPA buffer for harvesting and lysing cells, and the Bradford assay to measure protein concentrations. A 12% SDS-PAGE gel was used to separate whole-cell lysates. The separated proteins were transferred onto nitrocellulose membranes, blocked with 5% bovine serum albumin (BSA), and then washed twice with 0.5% TBS-Tween before being incubated with the primary antibody overnight at 4 °C. Subsequently, the membranes were washed five times with 0.5% TBS-Tween and then incubated for 2 h (room temperature) with anti-rabbit or anti-mouse IgG antibody conjugated with horseradish peroxidase (HRP) (depending on the primary antibody). The membranes were rewashed, and bound antibodies were detected using a chemiluminescent detection system, followed by visualization on an iBright™ CL-1500 imaging system (Thermo Fisher Scientific, Waltham, MA, USA). Protein loading consistency was verified by stripping the membranes and re-probing with an anti-β-actin antibody. IMAGE J software (version 1.410) was used to quantify band intensities.

### 2.9. Statistical Analysis

The data were statistically analyzed with GraphPad Prism software (version 8.0) based on three independent experiments using the independent samples *t*-test and Dunnett’s 1-way ANOVA. For data interpretation, cells treated with 0 μM of Loureirin A or Loureirin B were designated as the control group. Data from the control group were set to 100% to calculate the percentage of cell viability, migration, invasion, MMP activity, and protein expression in the treatment groups relative to the control (% of control). Similarly, the fold change of phosphorylated/total proteins in the control group was set to 1 to determine the relative fold change in the treatment groups. Before analysis, data normality was assessed using the Shapiro–Wilk test to confirm a normal distribution. Results were presented as mean ± standard deviation (mean ± S.D.). Statistical significance was defined as * *p* < 0.05, ** *p* < 0.01, and *** *p* < 0.001 compared to control (0 µM).

## 3. Results

### 3.1. Effect of Loureirin A and Loureirin B on Human NSCLC Cell Viability

**Figure 1:**
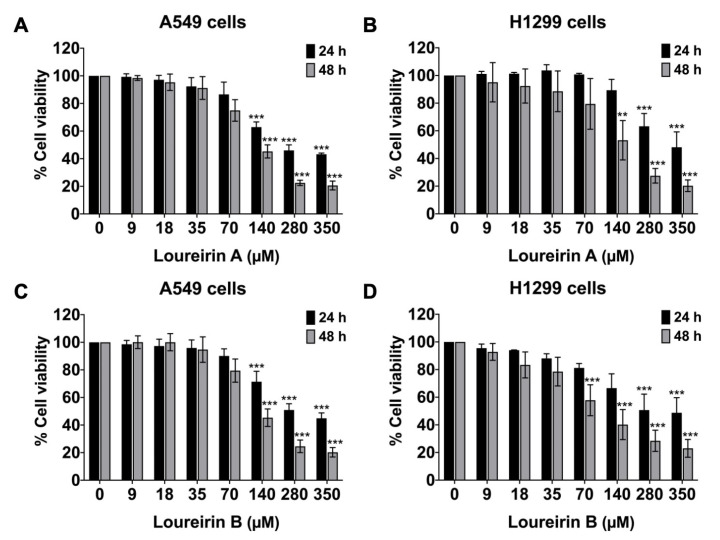
The impacts of Loureirin A and Loureirin B on the viability of A549 and H1299 cells. The viabilities of A549 cells (**A**,**C**) and H1299 cells (**B**,**D**) were assessed following treatment with Loureirin A and Loureirin B at varying concentrations from 0 to 350 µM for 24 h and 48 h using the SRB assay. Cells treated with 0 µM of Loureirin A or Loureirin B are presented as 100% of the control. Data are presented as mean ± SD value obtained from three independent experiments. ** *p* < 0.01, and *** *p* < 0.001 compared with control group (0 µM).

To evaluate the anti-migration and anti-invasion potential of Loureirin A and Loureirin B, we first assessed their effects on the viability of A549 and H1299 cells using the sulforhodamine (SRB) assay. The cells were treated with increasing concentrations of Loureirin A and Loureirin B (0, 9, 18, 35, 70, 140, 280, and 350 μM) for 24 and 48 h. The impacts of Loureirin A and Loureirin B on the viability of the A549 and H1299 cells are displayed in Figure 1. Based on these results, the IC_50_ (50% inhibitory concentration) values are presented in Table 1. The IC_50_ values for Loureirin A were determined to be 245 μM and 133 μM for the A549 cells and 280 μM and 126 μM for the H1299 cells after 24 and 48 h of treatment, respectively. Similarly, the IC_50_ values for Loureirin B were found to be 350 μM and 150 μM for the A549 cells and 280 μM and 136 μM for the H1299 cells after 24 and 48 h of treatment, respectively. Notably, concentrations ranging from 0 to 140 μM were non-toxic to both A549 and H1299 cells after 24 h of treatment, as they maintained cell viabilities above 80% (Figure 1). These findings guided the selection of concentrations up to 140 μM for 24 h treatments in subsequent experiments to evaluate their anti-migration and anti-invasion properties.

### 3.2. Evaluation Loureirin A and Loureirin B Induced Toxicity in Normal Cells

**Figure 2:**
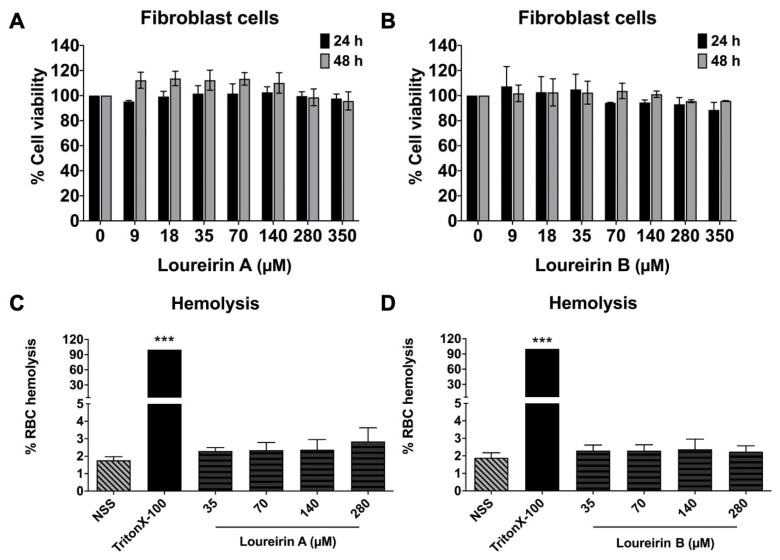
The impacts of Loureirin A and Loureirin B on the viability of primary human dermal fibroblast cells and red blood cells (RBCs). Fibroblast viability was assessed using the SRB assay after treatment with Loureirin A (**A**) and Loureirin B (**B**) at 0–350 µM for 24 and 48 h. RBC hemolysis was measured for Loureirin A (**C**) and Loureirin B (**D**), with NSS as the negative control and 0.5% Triton X-100 as the positive control (set as 100%). Data are presented as mean ± SD value obtained from three independent experiments. *** *p* < 0.001 compared to a negative control (NSS).

Loureirin A and Loureirin B are flavonoid compounds that are affordable, eco-friendly, and exhibit minimal to no adverse effects. To assess their safety, we evaluated their cytotoxicity in normal primary human dermal fibroblast cells. The results showed that Loureirin A (Figure 2A) and Loureirin B (Figure 2B) exhibited no cytotoxic effects on primary human dermal fibroblast cells, even after 24 or 48 h of treatment at the concentrations used in the anti-migration and anti-invasion studies. Additionally, Loureirin A and Loureirin B, at concentrations ranging from 0 to 280 μM, did not induce hemolysis in red blood cells (RBCs) (Figure 2C,D). These findings confirm that Loureirin A and Loureirin B are non-toxic and safe for use in normal cells.

### 3.3. Loureirin A and Loureirin B Impaired the Migration and Invasion Abilities of Human NSCLC Cells

**Figure 3:**
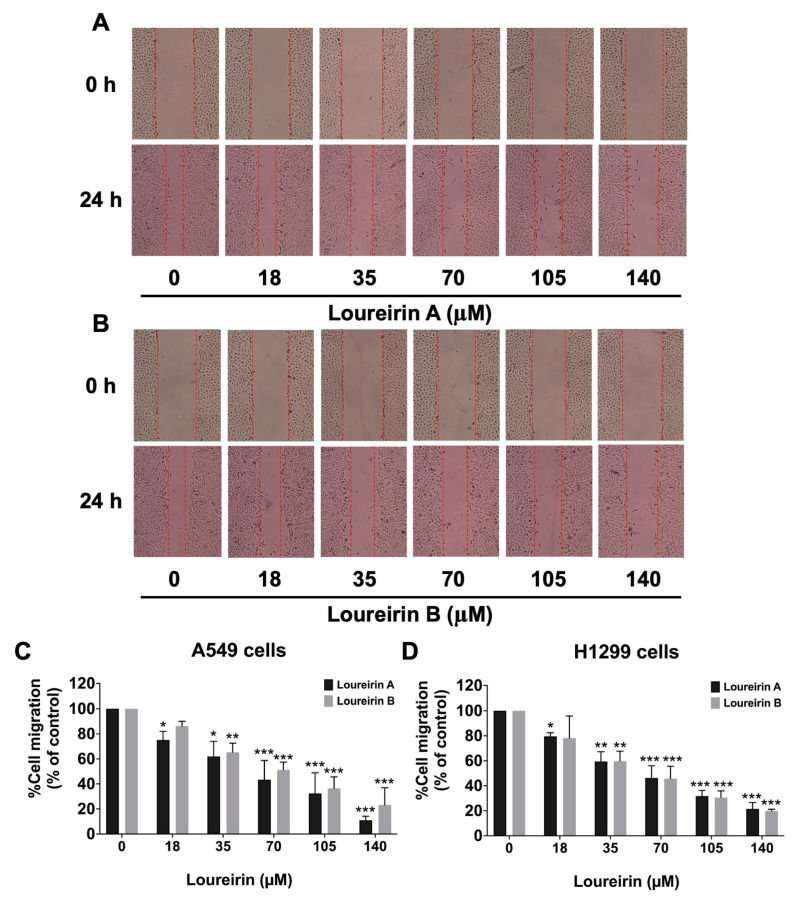
Loureirin A and Loureirin B suppressed A549 and H1299 cell migration in a wound-healing assay. Cells were treated with 0–140 µM Loureirin A or B for 24 h. Phase-contrast micrographs (×10) show scratch-wound distances in A549 cells treated with Loureirin A (**A**) and Loureirin B (**B**), while H1299 cell data are in Supplementary Figure S1. Quantitative analysis of A549 (**C**) and H1299 (**D**) cells is presented, with control (0 µM) set to 100% for migration calculations. Results are mean ± S.D. * *p* < 0.05, ** *p* < 0.01, *** *p* < 0.001 vs. control (0 µM).

**Figure 4:**
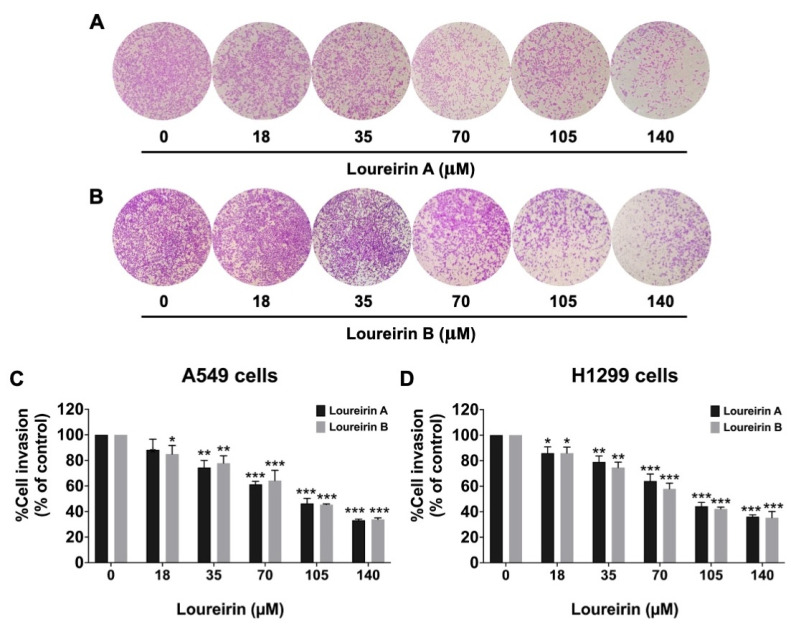
Loureirin A and Loureirin B suppressed A549 and H1299 cell invasion in Matrigel-coated trans-well assays. Cells were treated with 0–140 µM Loureirin A or B for 24 h. Phase-contrast images (×40) show the invaded A549 cells treated with Loureirin A (**A**) and Loureirin B (**B**), while H1299 cell data are in Supplementary Figure S2. Quantitative analysis of A549 (**C**) and H1299 (**D**) cells is presented, with control (0 µM) set to 100% for migration calculations. Results are mean ± S.D. * *p* < 0.05, ** *p* < 0.01, *** *p* < 0.001 vs. control (0 µM).

To evaluate the effects of Loureirin A and Loureirin B on the metastatic potential of NSCLC cells, their influence on the migratory and invasive capabilities of A549 and H1299 cells was analyzed using wound-healing and trans-well assays, respectively. As illustrated in Figure 3, Loureirin A significantly reduced the cell migration in both A549 (Figure 3A) and H1299 (Figure 3D and Figure S1A) cells in a dose-dependent manner (*p* < 0.001). Similarly, Loureirin B exhibited potent anti-migratory effects, also inhibiting migration in a dose-dependent manner in A549 (Figure 3B) and H1299 (Figure 3D and Figure S1B) cells (*p* < 0.001). Matrigel-coated trans-well assays were performed to further assess these compounds’ anti-invasion properties. Loureirin A markedly suppressed the invasive abilities of the A549 (Figure 4A) and H1299 (Figure 4D and Figure S2A) cell lines in a dose-dependent manner (*p* < 0.001). Similarly, Loureirin B also significantly inhibited both A549 (Figure 4B) and H1299 (Figure 4D and Figure S2B) cell invasion in a dose-dependent manner (*p* < 0.001). These findings reveal that both Loureirin A and Loureirin B possess strong anti-migratory and anti-invasive activities in A549 and H1299 cells. These results provided the basis for further investigation into the molecular mechanisms underlying their effects on NSCLC metastasis.

### 3.4. Loureirin A and Loureirin B Reduce MMP-2 and MMP-9 Activity and Suppress the Expression of Invasive Proteins in Human NSCLC Cells

**Figure 5:**
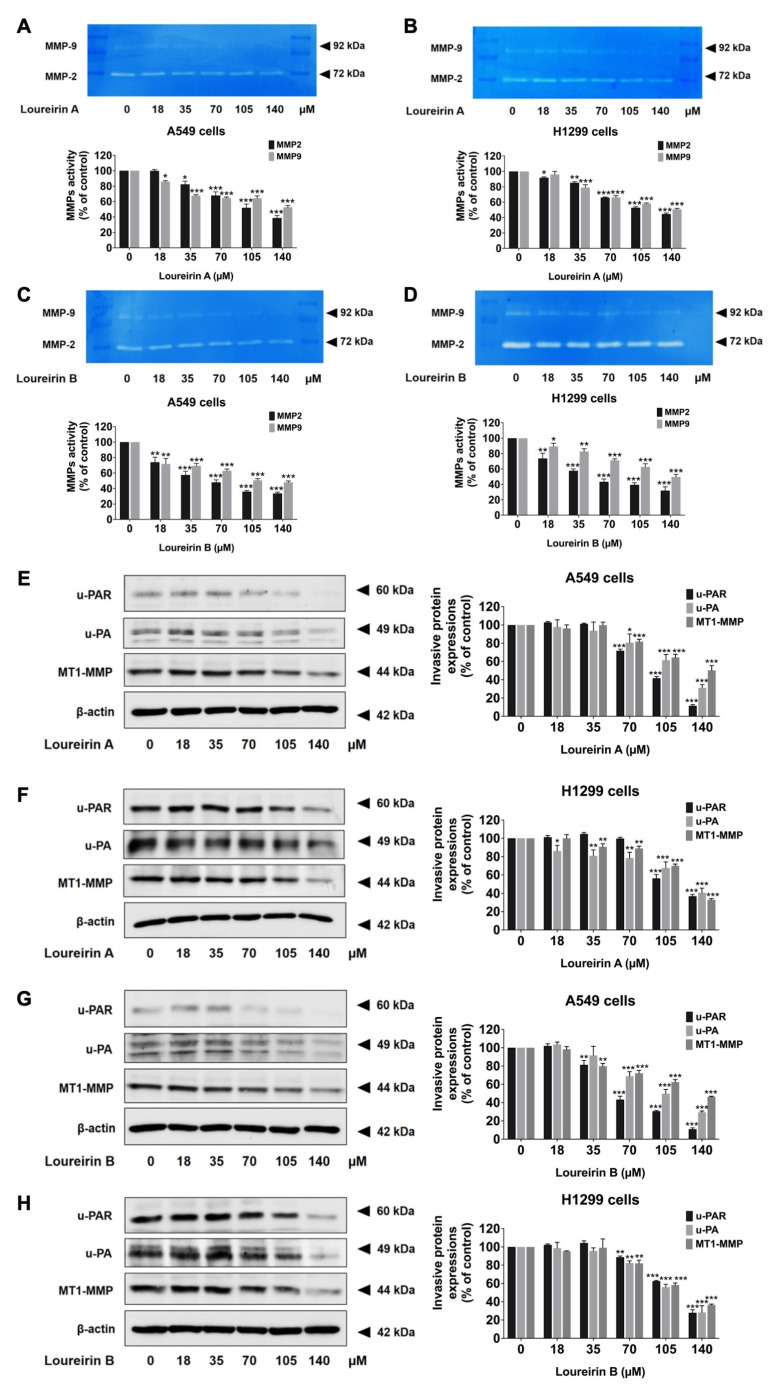
Loureirin A and Loureirin B reduced the levels of invasion-related proteins in NSCLC cells. A549 and H1299 cells were treated with 0–140 µM Loureirin A or B for 48 h. MMP-9 and MMP-2 activity was assessed via gelatin zymography (**A**–**D**), while u-PAR, u-PA, and MT1-MMP expression was analyzed by Western blotting (**E**–**H**). Data from the control group (0 µM) were set to 100% to calculate the percentage of protein expression. Results are expressed as mean ± S.D. * *p* < 0.05, ** *p* < 0.01, and *** *p* < 0.001 compared with a control group (0 µM).

To elucidate the molecular mechanisms by which Loureirin A and Loureirin B inhibit migration and invasion in NSCLC cells, we analyzed the activity of matrix metalloproteases (MMP-2 or gelatinase A and MMP-9 or gelatinase B) in culture supernatant using a gelatin zymography assay. The proteinase activity was visualized as clear bands in the polyacrylamide gel at 92 kDa and 72 kDa, corresponding to MMP-9 and MMP-2, respectively (Figure 4). Loureirin A significantly reduced the MMP-9 activity (*p* < 0.001; Figure 5A,B), while Loureirin B effectively suppressed the MMP-2 activity (*p* < 0.001; Figure 5C,D), which is consistent with the observed reduction in cell invasiveness for both A549 and H1299 cells.

Additionally, the expression levels of key invasive proteins, including u-PAR, u-PA, and MT1-MMP, were evaluated using Western blotting. Loureirin A significantly downregulated the expression of u-PAR, u-PA, and MT1-MMP in a dose-dependent manner (*p* < 0.001) in both A549 (Figure 5E) and H1299 (Figure 5F) cells. Similarly, Loureirin B markedly reduced the expression levels of these proteins in both A549 (Figure 5G) and H1299 (Figure 5H) cells. These results indicate that Loureirin A and Loureirin B effectively inhibit the activity of MMP-2 and MMP-9 and suppress the expression of u-PAR, u-PA, and MT1-MMP, thereby attenuating the invasive potential of human NSCLC cells.

### 3.5. Loureirin A and Loureirin B Decrease EMT Protein Levels in Human NSCLC Cells

**Figure 6:**
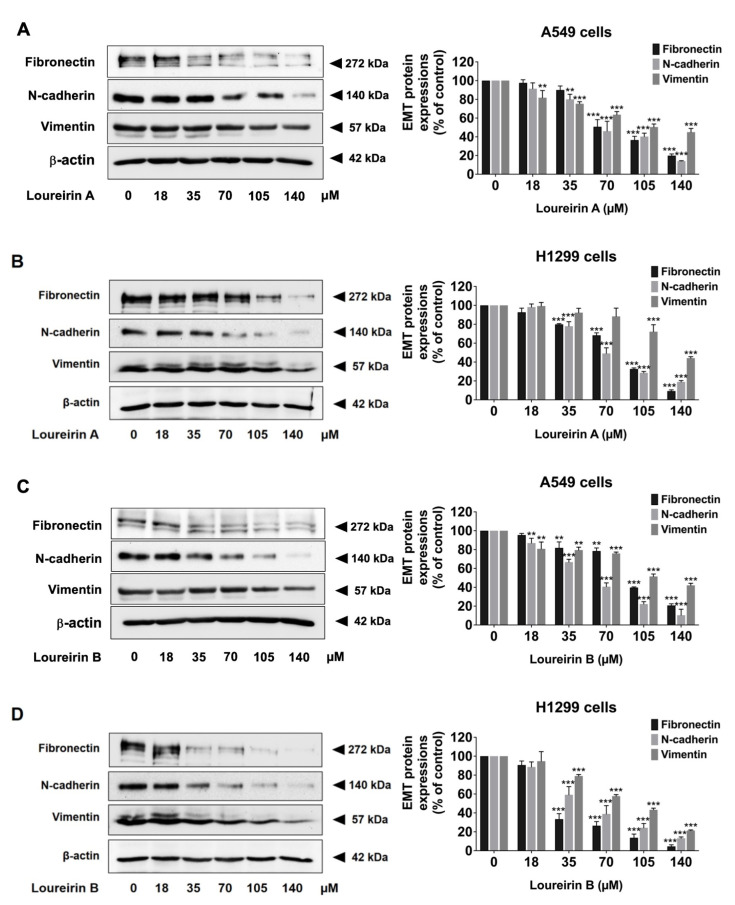
Loureirin A and Loureirin B suppressed the expression of EMT-associated markers of A549 and H1299 cells. A549 (**A**,**C**) and H1299 (**B**,**D**) cells were treated with 0–140 µM Loureirin A or B for 48 h. Fibronectin, N-cadherin, and vimentin expression were analyzed by Western blotting. Data from the control group (0 µM) were set to 100% to calculate the percentage of protein expression. Results are expressed as mean ± S.D. ** *p* < 0.01 and *** *p* < 0.001 compared with a control group (0 µM).

Epithelial–mesenchymal transition (EMT) is a critical biological mechanism in cancer metastasis, during which epithelial cells lose their polarity and adhesion properties, acquiring mesenchymal-like features [12]. EMT is characterized by changes in the expression of epithelial and mesenchymal markers, such as E-cadherin, vimentin, and N-cadherin. In this study, Western blot analysis demonstrated that Loureirin A significantly reduced the expression of mesenchymal markers, including fibronectin, N-cadherin, and vimentin (*p* < 0.001) in both A549 (Figure 6A) and H1299 (Figure 6B) cells. Similarly, Loureirin B markedly downregulated the expression of these markers (*p* < 0.001) in both A549 (Figure 6C) and H1299 (Figure 6D) cells. These findings suggest that Loureirin A and Loureirin B effectively suppress EMT, thereby attenuating the invasive potential of human NSCLC cells.

### 3.6. Loureirin A and Loureirin B Suppress the Activation of the MAPK Signaling Pathway in Human NSCLC Cells

**Figure 7:**
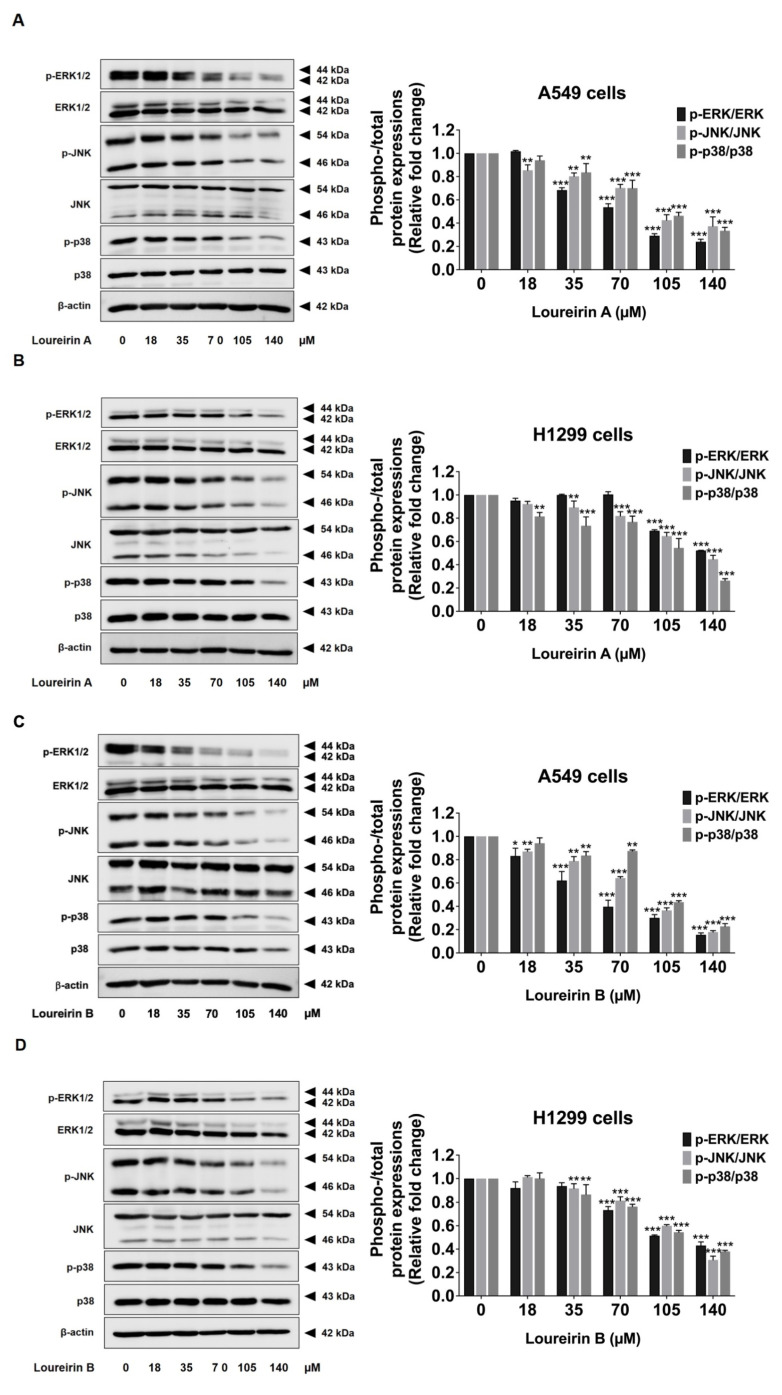
Loureirin A and Loureirin B suppressed the activation of the MAPK signaling pathway in A549 and H1299 cells. A549 (**A**,**C**) and H1299 (**B**,**D**) cells were treated with 0–140 µM Loureirin A or B for 48 h. Phosphorylation of ERK, JNK, and p38 was assessed by Western blotting. The fold change of phosphorylated/total proteins in the control group (0 µM) was set to 1 to determine the relative fold change in the treatment groups. Results are expressed as mean ± S.D. * *p* < 0.05, ** *p* < 0.01, and *** *p* < 0.001 compared with a control group (0 µM).

To further investigate the mechanisms through which Loureirin A and Loureirin B inhibit EMT and regulate the expression of invasive proteins, we examined their effects on the MAPK signaling pathway using Western blot analysis. We hypothesized that both compounds could suppress MAPK pathway activation in A549 and H1299 cells. The results revealed that Loureirin A significantly decreased the phosphorylation levels of ERK, JNK, and p38 in a dose-dependent manner (*p* < 0.001) in A549 (Figure 7A) and H1299 (Figure 7B) cells. Similarly, Loureirin B also inhibited the phosphorylation of ERK, JNK, and p38 in both A549 (Figure 7C) and H1299 (Figure 7D) cells. These findings confirm that Loureirin A and Loureirin B effectively suppress the MAPK signaling pathway, providing further insight into their roles in mitigating the invasive potential of human NSCLC cells.

## 4. Discussion

NSCLC remains one of the most aggressive and lethal malignancies worldwide, with increasing incidence and mortality rates underscoring the need for novel and effective therapeutic strategies [4,5]. Despite advancements in treatment modalities, metastasis continues to be the leading cause of cancer-related deaths, contributing to poor patient outcomes [6]. Targeting the metastasis cascade is, therefore, critical for improving the prognosis and survival in NSCLC patients [42,43,44]. Natural compounds derived from medicinal plants, such as flavonoids, have gained significant attention for their ability to modulate key oncogenic pathways and inhibit cancer progression [45,46].

Loureirin A and Loureirin B, two principal flavonoids extracted from the Thai traditional plant *Dracaena loureiri* [47,48], have previously been reported to exhibit anti-proliferative effects by inducing cell-cycle arrest at the G0/G1 phase and promoting apoptosis in NSCLC cells [49]. Building on these findings, the present study provides novel insights into their anti-metastatic potential. Both compounds demonstrated the ability to suppress epithelial–mesenchymal transition (EMT), a hallmark of cancer metastasis. Loureirin A and Loureirin B significantly suppressed the expression of mesenchymal markers (N-cadherin, vimentin, and fibronectin) while restoring epithelial marker expression, which is indicative of EMT inhibition. This finding supports previous reports that targeting EMT is a promising approach to impeding metastasis in various cancer types [50].

Furthermore, our results showed that Loureirin A and Loureirin B effectively attenuated the activity of matrix metalloproteinases (MMP-2 and MMP-9), as well as the expression of invasive proteins, including uPA, uPAR, and MT1-MMP. These proteins are essential mediators of extracellular matrix degradation, facilitating cancer cell invasion and dissemination [15,16]. The observed downregulation of these factors corroborates the anti-invasive properties of Loureirin A and Loureirin B, aligning with other studies that highlight the importance of modulating MMP activity in metastatic suppression [51,52].

An important aspect of evaluating potential therapeutic agents is their safety for normal cells. In this study, Loureirin A and Loureirin B exhibited no cytotoxic effects on primary human dermal fibroblast cells, even after 24 and 48 h of treatment at the concentrations used in the anti-migration and anti-invasion studies. Additionally, both compounds demonstrated minimal to no hemolytic activity on human red blood cells (RBCs), even at concentrations as high as 280 mM. These findings underscore the safety profiles of Loureirin A and Loureirin B and support the potential for their use in therapeutic applications without adverse effects on normal tissues.

**Figure 8:**
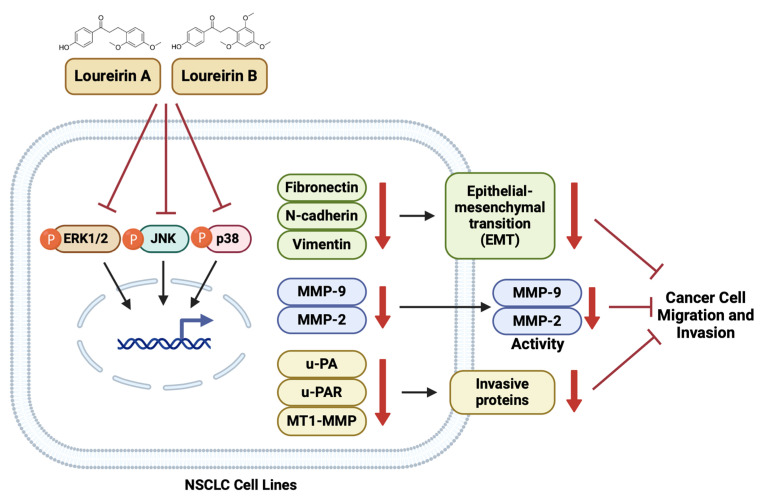
A schematic summarizing the mechanism by which Loureirins A and B from *Dracaena loureiri* inhibit EMT and invasion in NSCLC cell lines.

Mechanistically, we identified the MAPK signaling pathway as a key target of Loureirin A and Loureirin B. Previous studies have reported that the constitutive activation or dysregulation of MAPK signaling pathways is implicated in the initiation, progression, and metastatic spread of lung cancer [53]. The p38 MAPK pathway regulates cell invasion, migration, and EMT in lung adenocarcinoma [54]. Additionally, the ERK/JNK/p38 MAPK signaling pathways contribute to the progression and metastasis of NSCLC cells [55]. Aberrant activation of the MAPK signaling pathway is a characteristic feature of various cancers, including NSCLC, where it promotes EMT, cell migration, and invasion [18,56,57]. Our study demonstrated that both compounds significantly inhibited the phosphorylation of ERK, JNK, and p38, the three major MAPK subfamilies, thereby disrupting this pathway. Figure 8 presents a schematic summary of the mechanism by which Loureirins A and B inhibit EMT, migration, and invasion in NSCLC cell lines. In addition to their impact on MAPK signaling, Loureirin A and Loureirin B may have broader implications for NSCLC treatment. Previous studies have shown that Loureirin A and Loureirin B affect multiple signaling pathways involved in cell metastasis. In various cell systems, Loureirin A has been shown to suppress MMP-9 in mouse articular chondrocytes by inhibiting the Akt/NF-κB signaling pathway [28], to promote melanoma cell differentiation, and to suppress migration and invasion by inhibiting the WNT and AKT/mTOR signaling pathways [58]. Similarly, Loureirin B has been reported to reduce MMP-2 and MMP-9 levels and inhibit HeLa cell metastasis through inhibition of the PI3K/AKT signaling pathway [59]. However, our findings highlight the potential of Loureirin A and Loureirin B in modulating cancer progression in NSCLC through the MAPK signaling pathway. Inflammation plays a pivotal role in the metastatic cascade by fostering a tumor-promoting microenvironment [60,61]. Although not directly examined in this study, the known anti-inflammatory properties of Loureirin A and Loureirin B suggest their potential to mitigate inflammatory process that contributes to NSCLC progression. Loureirins A and B, due to their potent anti-invasion and anti-cancer properties, hold potential as valuable components in combination therapy. Their ability to inhibit key signaling pathways, such as the MAPK pathway, and suppress EMT-associated markers may enhance the efficacy of chemotherapy or improve responses to targeted therapies. However, further investigations should explore this aspect to provide a more holistic understanding of their therapeutic benefits.

Despite these promising findings, certain limitations of our study should be acknowledged. First, all experiments were conducted in vitro using A549 and H1299 cell lines, which may not fully replicate the complexity of metastatic behavior in vivo. While Loureirin A and Loureirin B show potential in modulating multiple signaling pathways involved in cancer progression, including the MAPK, PI3K/AKT, and Wnt/β-catenin pathways, compounds that affect multiple pathways are often metabolized rapidly. This rapid metabolism limits the bioavailability of Loureirin A and Loureirin B, thereby reducing their efficacy in in vivo studies and presenting a significant pharmacokinetic challenge. Consequently, these compounds may exhibit only modest activity, requiring higher concentrations compared to other anticancer drugs. Therefore, further studies utilizing animal models are necessary to confirm their anti-metastatic effects under more complex physiological conditions. Additionally, while this study focused on the MAPK pathway, other signaling pathways implicated in NSCLC metastasis, such as the PI3K/AKT, NF-κB, and Wnt/B-catenin pathways, should be investigated to comprehensively elucidate the mechanism of action of these compounds. Lastly, pharmacokinetic and pharmacodynamic studies are necessary to evaluate the bioavailability, efficacy, and safety of Loureirin A and Loureirin B in their potential clinical application.

In conclusion, our findings demonstrate that Loureirin A and Loureirin B exhibit potent anti-migration and anti-invasion properties by suppressing EMT, reducing the activity of MMPs activity and the expression of invasive proteins, and inhibiting MAPK signaling. Furthermore, their non-toxic nature, evidenced by their lack of cytotoxicity in human dermal fibroblasts and their hemolytic activity in RBCs, highlights their potential as safe therapeutic agents. Although Loureirin A and Loureirin B show significant anti-migration and anti-invasion effects in vitro, as noted in the limitations section, both compounds may undergo rapid transformation. Consequently, they require higher concentrations compared to other anti-cancer drugs. Future studies addressing the limitations of these compounds and exploring combination therapies may pave the way for their translation into clinical practice.

## Figures and Tables

**Table 1:** The IC_50_ values of Loureirin A and Loureirin B in NSCLC cell lines.

		A549 Cells	H1299 Cells
Loureirin A (µM)	24 h	245 ± 22.91	280 ± 21.79
	48 h	133 ± 8.19	126 ± 9.07
Loureirin B (µM)	24 h	350 ± 13.23	280 ± 20.00
	48 h	150 ± 15.00	136 ± 19.66

## Data Availability

Data are contained within the article and Supplementary Materials.

## Supplementary Materials

The following supporting information can be downloaded at: https://www.mdpi.com/article/10.3390/life15030396/s1, Figure S1: The impact of Loureirin A and Loureirin B suppressed the migration on H1299 cells using a wound healing assay.; Figure S2: The impact of Loureirin A and Loureirin B suppressed the invasion on H1299 cells using Matrigel-coated Trans-well assays.
