# Supplementary material for: Targeting MAPK Signaling: Loureirins A and B from Dracaena Loureiri Inhibit Epithelial–Mesenchymal Transition and Invasion in Non-Small Cell Lung Cancer Cell Lines
Source: Life (Basel). 2025 Mar 3;15(3):396. doi: 10.3390/life15030396 (PMC11943645; doi:10.3390/life15030396)
Supplement: Supplementary file 1 [file life-15-00396-s001.zip › life-3474501-supplementary.pdf]

Supplement data

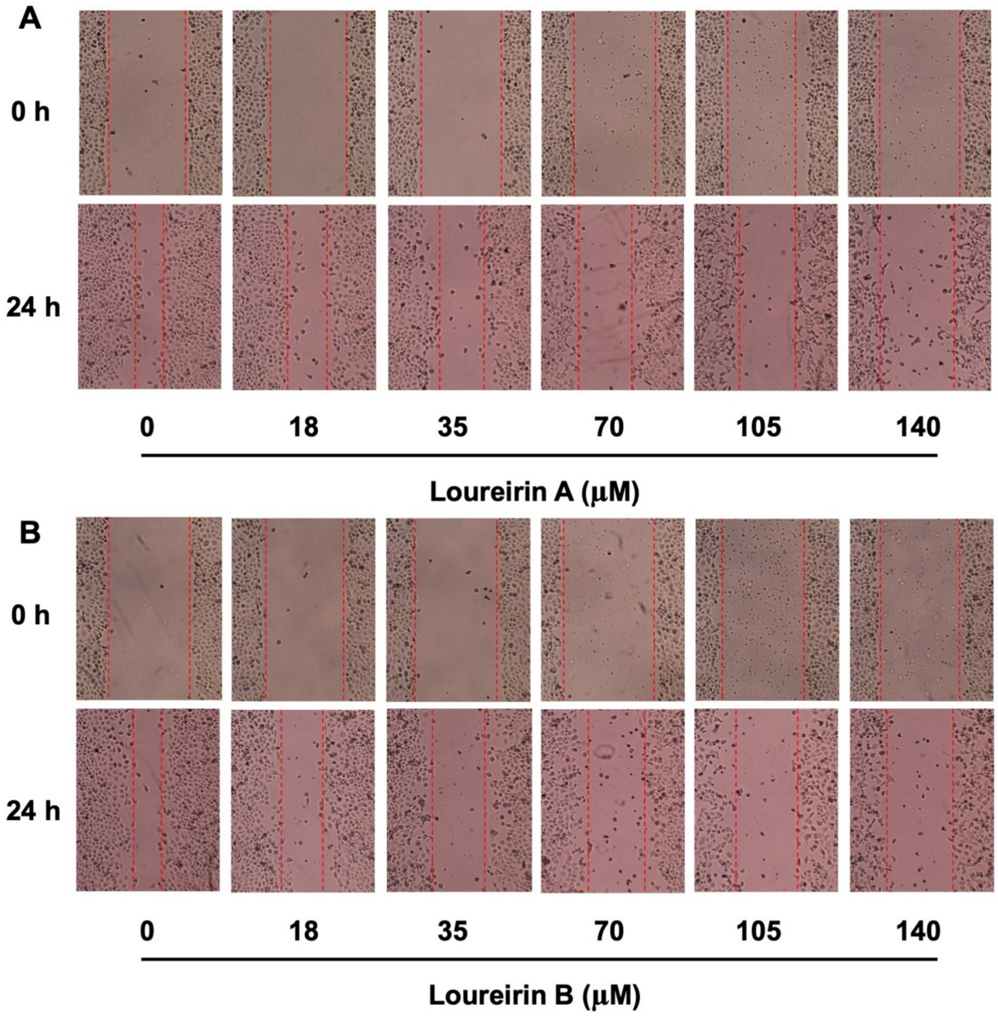

Figure S1. The impact of Loureirin A and Loureirin B suppressed the migration on H1299 cells using a wound healing assay. H1299 cells were treated with Loureirin A (A) and Loureirin B (B) at the concentrations of 0-140 µM for 48 hours were subjected to wound healing assays. The invading cells were visualized using phase-contrast microscopy and analyzed using ImageJ software.

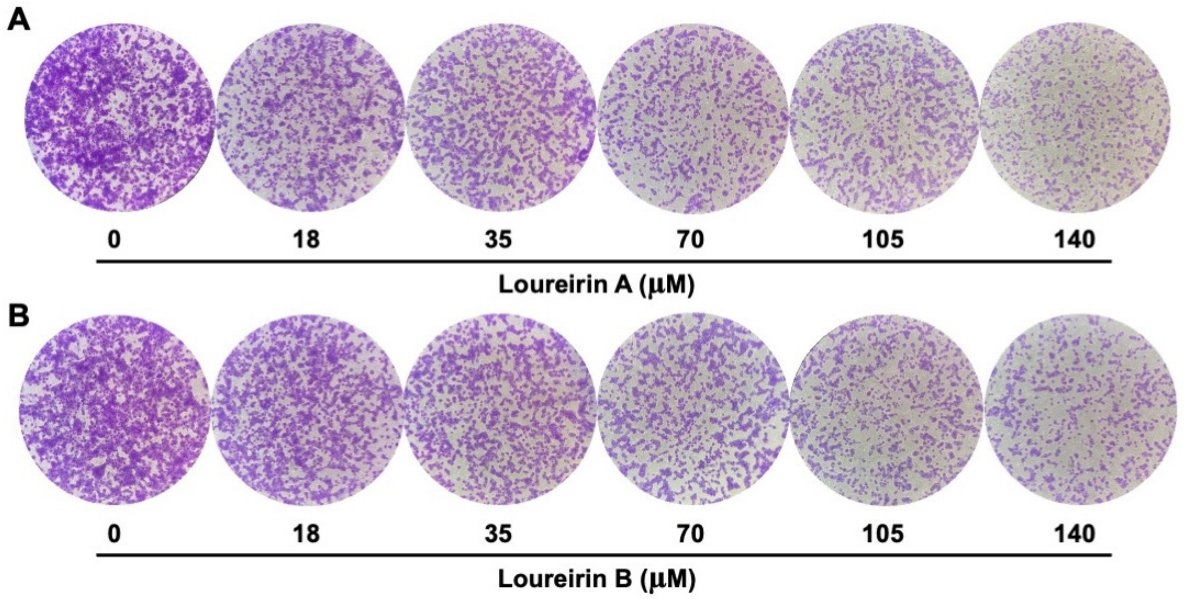

Figure S2. The impact of Loureirin A and Loureirin B suppressed the invasion on H1299 cells using Matrigel-coated Trans-well assays. H1299 cells were treated with Loureirin A (A) and Loureirin B (B) at concentrations of 0-140 μM for 24 hours were subjected to Trans-well invasion assays. The invading cells were visualized using phase-contrast microscopy and analyzed using ImageJ software.
